# Suckling and allosuckling behavior of dairy calves in indoor dam-rearing systems

**DOI:** 10.3389/fvets.2025.1617158

**Published:** 2025-07-17

**Authors:** Claire S. Wegner, Cady W. Chan, Lars Rönnegård, Sigrid Agenäs, Lena Lidfors, Hanna K. Eriksson

**Affiliations:** ^1^Department of Applied Animal Science and Welfare, Swedish University of Agricultural Sciences, Uppsala, Sweden; ^2^Department of Animal Biosciences, Swedish University of Agricultural Sciences, Uppsala, Sweden; ^3^School of Information and Engineering, Dalarna University, Falun, Sweden

**Keywords:** cross-suckling, calf management, voluntary milking system, dam-rearing, cow-calf contact

## Abstract

An important element in dairy cow-calf contact (CCC) systems is to ensure sufficient milk intake by calves. However, little is known about possible changes in suckling behavior during suckling periods for calves up to 15 weeks old, and the prevalence of allosuckling is poorly understood in the context of these systems. This research had two aims: first, to explore possible changes in suckling behavior as calves aged when housed in an indoor CCC system, and second, to identify calf-level factors associated with allosuckling. Both aims were independently investigated in two separate studies (cow- and calf-driven contact, respectively) and involved both Swedish Red and Swedish Holstein dams and calves. In the cow-driven study, dam-calf pairs (*n* = 19 male and female calves) had shared access to a separate contact area containing stalls, which dams could leave at any time. In the calf-driven study, calves (*n* = 24 female calves) could access their dams (*n* = 23) in all parts of the pen, except the milking area. Behavior sampling from video was used to record suckling behaviors during a 24-h period at average calf ages of 3, 6, 9, 12 (both studies) and 15 (cow-driven only) weeks. In the cow-driven study, calves behaved consistently across all weeks in terms of suckling bout length and frequency. Calves in the calf-driven study took significantly fewer, but longer, suckling bouts as they aged. The overall frequency of allosuckling observed in the cow-driven study (36%) was higher than that in the calf-driven study (14%). However, the odds of allosuckling increased significantly with increasing calf age in both studies. Calves in the cow-driven study were observed to allosuckle even in the presence of their own dam, and increasingly so as they aged. For both studies, instances of allosuckling were over 140 times more likely when other calves were already engaged in suckling on a cow. We conclude that allosuckling is likely to occur in indoor dam-rearing systems when the animals are housed in automatic milking systems, although the frequency will depend on the age of the calves and the presence of other suckling calves.

## 1 Introduction

In intensive dairy production systems, calves are most commonly separated from the dam within hours of being born and then reared artificially, leaving them with limited opportunities to exhibit suckling behavior. Calves are highly motivated to suckle and, when prevented from performing this behavior (e.g., feeding via automatic feeders), have been shown to develop non-nutritive oral behaviors (1). From studies performed under semi-natural conditions, it is known that calves of beef (*Bos taurus*) and Zebu (*Bos indicus*) dairy breeds that are reared by their dams will perform between 9 and 11 suckling bouts within a 24-h period when they are younger than 3 weeks (2, 3), with individual bouts lasting approximately 10–12 min (4, 5). This behavior has been observed to change as calves age, particularly during the first few months, with fewer – but longer – suckling bouts performed (3, 6, 7). Similar patterns of behavioral change have been noted for dam-reared beef calves in confined housing systems (8, 9) and Zebu dairy calves in restricted suckling systems (i.e., 30 min of dam-calf contact twice daily) where cows were also milked (10).

When dairy calves are housed in cow-calf contact (CCC) systems instead of being reared artificially, they will have opportunities to suckle and engage in pre- and post-stimulation behaviors, more closely reflecting the situation under semi-natural conditions and in beef production. Interest toward CCC systems is growing, as evidenced by the recommendations for increased implementation of prolonged (i.e., >24 h) CCC outlined in a recent European report on calf welfare (11). In these systems, dairy calves are housed together with lactating dairy cows, although the type of CCC [i.e., full or partial physical contact; dam or foster cow; (12)] and duration of daily contact permitted can vary greatly between system setups [for variation in European countries, see survey study by Eriksson et al. (13)].

To date, suckling behavior has been described for a variety of CCC systems, including indoor freestall dam-rearing systems (14, 15). There is some evidence to suggest that dairy calves, similar to that which we described earlier for calves under semi-natural conditions, change their behavior to perform fewer (14, 16) – but longer (17) – suckling bouts as they age. However, observations have previously been limited to 9 weeks of age, which is still short of the weaning age range currently reported for European CCC systems (median: 12–17 weeks) (13).

The first aim of our research was to explore how suckling behavior – including suckling bout duration, bout frequency, and the total time per day spent suckling – changed with age for dairy calves housed in indoor CCC systems with either cow- or calf-driven contact with dams. Whether the system was considered cow- or calf-driven depended on which individuals (i.e., dams or calves) could take primary initiative of CCC within the pen [see Sirovnik et al. (12) for detailed definitions]. The ages studied (cow-driven study: 3–15 weeks, calf-driven study: 3–12 weeks) may offer insight into calf behavior during a suckling period that better represents that of current practices, therefore increasing our knowledge base for future management recommendations.

Additionally, while allosuckling (i.e., the act of suckling from an alien cow) has previously been reported for CCC systems with dam-calf contact, observations of the behavior in calves have either been evaluated at only two points in early life (16, 18), or summarized across multiple ages (14). In general, our current understanding of allosuckling in dairy calves is limited, in terms of how it is affected both by calf age and the housing system (e.g., if the calves have access to parts of or the whole pen). Our second aim was therefore to identify potential calf-level factors associated with allosuckling in dairy calves housed in these two different CCC systems. We further wanted to describe the overall frequency of allosuckling in both systems, although any comparisons between systems will be purely descriptive as the study set-up differed in multiple ways. Finally, there are certain characteristics that may differ between bouts of allosuckling and suckling bouts on the dam. For example, it has been suggested that allosuckling primarily occurs in positions that allow the calf to avoid identification by the cow through smelling or ano-genital licking (19). As such, we also sought to descriptively present calf position and the occurrence of allogrooming during suckling events.

## 2 Materials and methods

### 2.1 Animals, housing and management

Both of the studies described below were conducted at the Swedish Livestock Research Centre in Uppsala, Sweden, and operated with full, whole-day CCC, where contact between dams and calves was possible at any point during the day apart from milking sessions. The sample sizes were based on the number of CCC cows recruited for two larger randomized control trials, also including conventionally kept cows not used in the current studies.

#### 2.1.1 Study 1: cow-driven CCC system

A total of 21 dam-calf pairs were enrolled for Study 1 (hereafter referred to as “cow-driven study”), which took place between October 2020 and January 2021. Dam-calf pairs were enrolled over a 6-week period and included both male and female calves. Dams (primiparous: *n* = 12, multiparous: *n* = 9) were only eligible for enrolment if they had no prior history of *S. aureus* mastitis (if multiparous) and were not severely lame [i.e., a gait score of 4 or 5, following Flower and Weary (20)] during the dry period, as per criteria that was established *a priori*. Pairs spent an average (SD) of 3 (0.6) days together in individual calving pens, located in a separate area, before being introduced to group housing in the experimental pen within the cow barn. Two of the 21 dam-calf pairs were removed from the study during the enrolment period – one due to euthanasia of the calf following a trauma (calf age: 30 days), and another after the dam died of *E. coli* mastitis (calf age: 11 days). The remaining calves were an average of 24 (12.6) days old when the study period began. The final number of dam-calf pairs present for observations during the study period – which lasted until an average calf age of 15 weeks – was 19 (Swedish Holstein [SH]: *n* = 7, Swedish Red [SR]: *n* = 12), including 7 male calves and 12 female calves.

**Figure 1:**
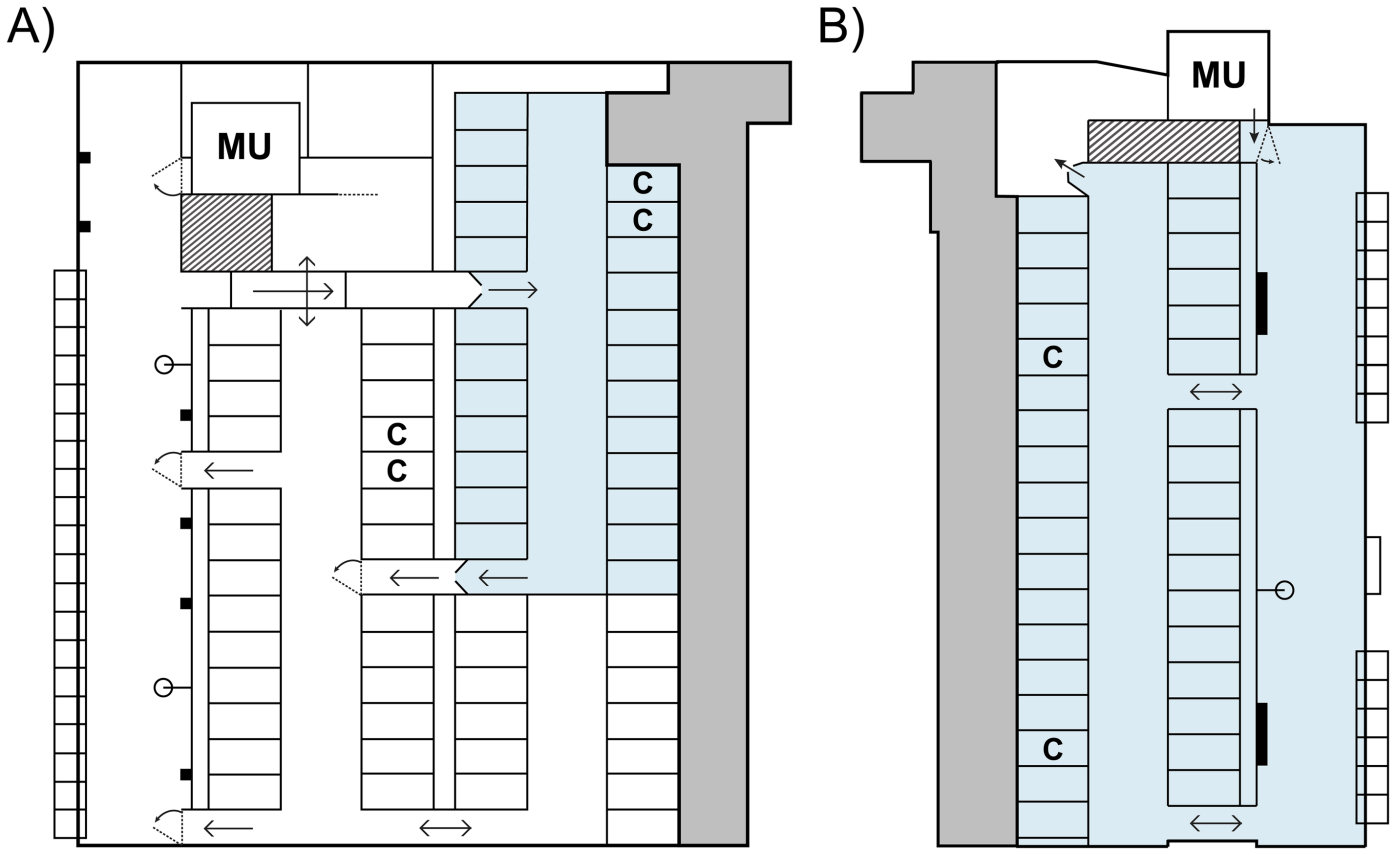
Schematic of the experimental pens used in the cow-driven **(A)** and calf-driven **(B)** systems, with areas permitting full CCC shown in blue. In the cow-driven system, contact between dam-calf pairs was only possible when cows spent time in the contact area, which they could freely leave. In the calf-driven system, calves could access their dams in almost all areas of the pen. Calves in both systems had additional, exclusive access to a separate calf creep (shown in dark grey), wherein they had access to roughage, concentrate, minerals, and water. All areas shown in white were only accessible to cows; spring-loaded one-way gates prevented calves from entering. MU = milking unit; C = concentrate feeding station. Figures are modified from Wegner and Ternman (21) and Wegner et al. (22).

Both dams and calves were housed in an indoor freestall pen stocked with 54 (3) cows during the study period that operated with a Feed First™ system (DeLaval International AB, Tumba, Sweden) and automatic milking (see Figure 1A). All cows, including the non-experimental cows, had shared access to two concentrate stations (DeLaval feed station FSC400, DeLaval International AB, Tumba, Sweden), 37 freestalls, a feed alley containing 20 individual feed bins (CRFI, BioControl AS, Rakkestad, Norway) and seven water bowls, and a milking area containing a waiting area and milking unit (DeLaval VMS™ Classic, DeLaval International AB, Tumba, Sweden). Contact between dams and calves was only possible in the contact area, which was an enclosed area within the experimental pen. Only dams with calves (i.e., enrolled in the study) had access to this area, which was controlled by an automatic selection gate (DeLaval Smart Selection Gate SSG, DeLaval International AB, Tumba, Sweden) when cows exited the feed alley. The contact area contained 22 shared freestalls, as well as two additional concentrate feeding stations for cows. Dams were directed to the milking area via the selection gate if more than 6 h had passed since their previous milking session. During the study period, cows were milked on average 2.3 (0.58) times per day and delivered 19.5 (9.34) kg of milk daily to the milking unit. As the dams could choose to leave the contact area when motivated to do so, they were the individuals primarily in control over how much dam-calf contact was possible in this study; we therefore refer to this CCC system as cow-driven.

Calves also had exclusive access to a 73.2 m^2^ deep-bedded calf creep containing water, roughage, and concentrate. Movement between the calf creep and contact area was possible through the fronts of the stalls, by walking under the neck and front rails. Spring-loaded one-way gates at both the entrance and exit of the contact area prevented calves from entering other parts of the pen. For more details on housing and management of dams and calves, see Wegner and Ternman (21).

#### 2.1.2 Study 2: calf-driven CCC system

Study 2 (hereafter referred to as “calf-driven study”) was carried out from March to May 2022 and involved an initial 24 dam-calf pairs and 1 dam-calf triad containing twin calves. Dams (primiparous: *n* = 11, multiparous: *n* = 14) and calves were enrolled over a 6-week period according to *a priori*-established enrolment criteria, which stated that the calf was female, and that the dam had no previous history of *S. aureus* mastitis (if multiparous) and was not severely lame during the dry period (following the same criteria as in the cow-driven study). Dam-calf units (SH: *n* = 9, SR: *n* = 16) were housed in individual calving pens for an average of 4 (1.0) days, after which they were introduced to the experimental pen in the cow barn. Calves were an average of 22 (11.4) days old when all pairs had entered the pen and the study period began. One SR dam-calf pair was removed from the study after the dam was diagnosed with and died of *E. coli* mastitis (calf age: 66 days), while another SH pair was removed due to congenital impaired digestive functioning of the calf (calf age: 87 days). The study period lasted until an average calf age of 12 weeks and ended in mid-May, when dams and calves were granted additional access to an outdoor pasture. A total of 23 dams and 24 calves were available for analyses.

Dams and calves were housed together in an indoor freestall pen with free cow traffic and automatic milking (Figure 1B); no other animals were housed in this pen. Within the pen, CCC was calf-driven, as calves were the primary initiators of contact in this system and could do so in all areas, apart from the calf creep, waiting area, and milking unit (DeLaval VMS™ V300, DeLaval AB International, Tumba, Sweden). Dams could enter the milking unit freely, and either be milked if they had milking permission (which was set at 6 h post-previous milking) or receive a portion of concentrate. On average during the study period, dams were milked 2.8 (0.62) times per day and delivered 18.7 (12.39) kg of milk daily to the milking unit.

Resources shared by both dams and calves included 33 freestalls, two self-filling water troughs, a swinging cow brush (DeLaval SCB, DeLaval International AB, Tumba, Sweden) and a small feeding table containing eight headlock spaces and 1.9 m of open feeding space, where feed was placed in a raised trough to be accessible for calves. Dams had additional access to 14 individual feed bins (CRFI, BioControl AS, Rakkestad, Norway). Meanwhile, calves also had exclusive access to an 80 m^2^ deep-bedded calf creep, which contained *ad libitum* access to water, concentrate and roughage. General pen design and management for this study are described in further detail in Wegner et al. (22).

### 2.2 Behavioral recordings

A total of eight (cow-driven study) and six (calf-driven study) fisheye cameras (Samsung SNF-8010VM, Samsung Techwin Co., Ltd., Seoul, South Korea) were installed overhead in all indoor areas. Dams were marked with animal-safe marking spray, while calves were fitted with colored collars to allow the identification of individuals. Behavioral observations were performed by three observers using video data at 24-h periods corresponding to average calf ages of 3, 6, 9, 12 (both studies) and 15 weeks (cow-driven study only). By default, observations occurred between 00:00 and 23:59 h; during three observation periods, adjustments to the start time were made to avoid periods with missing video data or major disturbances in the pen. Blinding the observers for study (cow-driven, calf-driven) or cow-calf relationship (dam vs. alien cow) was not possible as a result of the measures collected and methods used (i.e., video observations, where the entire pen was visible).

Continuous recording using behavior sampling (23) was used to record suckling bouts and close-to-udder events. The definitions for both behaviors were developed by the first and second authors following Fröberg and Lidfors (14) and tested using a 2-h subset of video data (hereafter referred to as the “training dataset”). The final definitions used for all data collection are as follows: a suckling bout was defined as the calf being near (<10 cm) or touching the udder with its mouth for ≥1 min and visibly, rhythmically sucking throughout. Contact between the mouth and udder could be broken for periods of <1 min, and suckling bouts that occurred within 10 min on the same cow were considered part of the same event (24, 25). Meanwhile, a close-to-udder event was defined as the calf being near (<10 cm) or touching the udder with its mouth, but with <1 min or no visible sucking activity. Close contacts that occurred <1 min apart and on the same cow were considered a single close-to-udder event.

The cow and calf ID were recorded for all behavioral events. If the event occurring was not between a dam-calf pair, it was additionally recorded if the focal calf’s dam was present (i.e., in a barn area accessible to the calf) upon initiation of the event. For both behaviors, event duration was calculated as the total time between first and last contact with the udder, including interruptions as permitted in the definitions.

**Figure 2:**
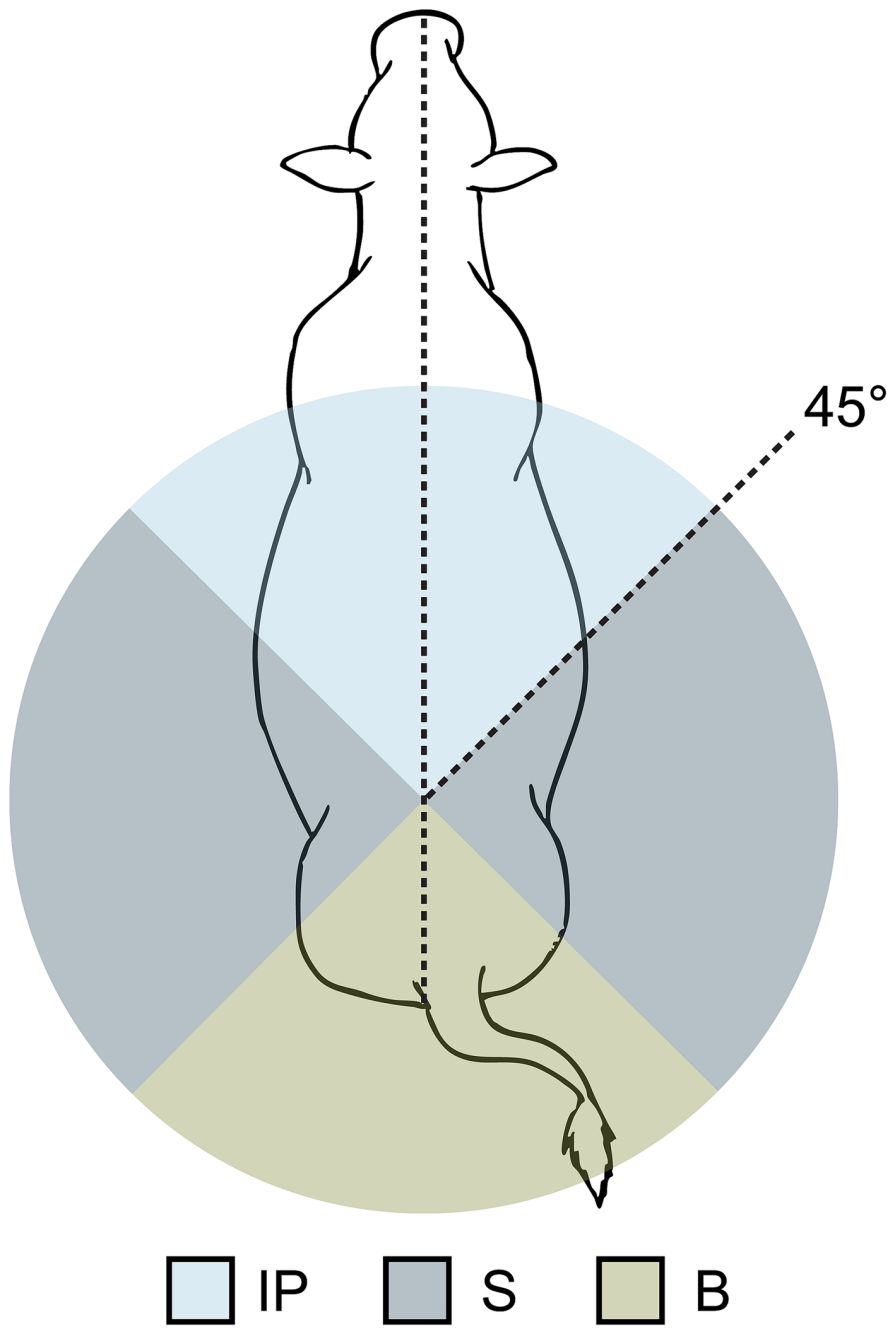
Schematic demonstrating the scoring of calf body position during suckling bouts, which was based on the angle of the calf’s body relative to the body of the cow. The position in which the calf spent the majority of a single suckling bout was recorded; possible positions included inverse parallel (IP), from the side (S), or from behind (B).

For suckling bouts, the primary body position of the calf relative to the cow was recorded as being inverse parallel (IP), from the side (S), or from behind (B) (Figure 2). As calf body angle relative to the cow was the only scoring factor, it was possible, for example, for a calf to suckle from between the hind legs but be scored ‘S’ for body position. Additionally, one–zero sampling was used to record allogrooming during, or within 1 min before or after, a suckling bout. Allogrooming was defined as licking between a focal cow and calf, and could be directed to any part of the recipient’s body. The individual(s) performing the licking (cow, calf or both) was not recorded.

The reason for termination of a suckling bout or close-to-udder event was additionally recorded as one of the following: (1) the focal calf walks or moves away, (2) the focal cow walks or moves away, (3) the focal cow kicks out or otherwise disrupts the bout (e.g., by butting or lunging at the calf, lying down or defecating), and (4) other. Reasons under ‘other’ included disruptions by non-focal animals, personnel or barn equipment (e.g., barn scrapers). Finally, it was binomially recorded (1 = yes, 0 = no) if at least one other calf was already engaged in suckling the focal cow when a suckling bout or close-to-udder event began. The conditions for scoring a ‘1’ included that a non-focal calf had to have a confirmed suckling bout of their own, and physically be in contact with the udder at the start time of the focal behavioral event.

#### 2.2.1 Inter-observer reliability

Following an initial training session, where a third observer was trained by the first and second authors (also observers) using the training dataset, all three observers performed independent behavioral recordings on video data from three separate days, covering a total 140 behavioral events. Each event was then scored binomially in terms of whether or not each observer recorded it, and the duration of each event (in seconds) was averaged across all observers. Initial visual analyses indicated that there was poor agreement between observers for very short events. This was confirmed when we performed initial statistical testing using the irr package (26) and calculated a Light’s kappa of 0.126. Using an iterative process, we determined that an appropriate cut-off for behavioral event duration was 16 s, as removing observations shorter than this resulted in the highest kappa coefficient (*κ* = 0.210) while eliminating as few “true” events as possible. While the kappa statistic itself indicates poor agreement, it is well known that a large difference in relative probability of an event occurring or not (indicated by a high prevalence index) results in paradoxically low kappa values (27). For the 74 events remaining after removing events shorter than 16 s, we obtained an overall agreement of 85% between raters (i.e., all three observers agreed on these events). The Prevalence Index (possible values −1 to 1; 0 indicates no difference in relative probability) and Bias Index (possible values −1 to 1; 0 indicates no bias between observers) were calculated for each pair of observers, resulting in a Prevalence Index ranging from 0.85 to 0.88 and a Bias Index ranging from −0.03 to −0.01. Combined, these metrics lead us to conclude sufficient inter-observer reliability for events 16 s or longer. To further reduce the risk of error in data recording, observers were instructed to flag uncertain events. These events were then reviewed with all observers present, and a consensus was reached.

### 2.3 Calf weight recordings

Calves in both studies were weighed at birth (mean (SD): cow-driven study = 38 (6.5) kg, calf-driven study = 40 (6.6) kg) and monthly thereafter throughout each study period. For calculations of average daily gain (ADG), we used birth body weight and the body weight collected in nearest proximity to the end of each study period. For the calf-driven study, this measure was collected 11 days before the end of the study period. Body weights used for the cow-driven study were, for practical reasons, collected on 2 separate days, corresponding to 1 and 4 days after the study period ended. ADG was calculated by subtracting birth weight from the body weight near the end of the study period and dividing by the difference in days between these two weighings.

### 2.4 Data handling and analysis

All data handling and statistical analyses were performed using R version 4.4.2 (28) and the tidyverse package (29). Statistical significance was accepted at *p* < 0.05. For all linear mixed effects models, test statistics and *p*-values were obtained using the car package (30) and following Al-Sarraj and Forkman’s (31) recommendations for analyzing unbalanced datasets. Results from linear mixed effects models were extracted using the emmeans package (32) and estimated responses are reported as LSMeans ± SEM. Raw data is presented as mean (SD) if normally distributed, while skewed data is reported as median and interquartile range (IQR). The individual calf was treated as the experimental unit in all analyses.

There were a total of 980 and 964 behavioral events recorded for the cow- and calf-driven studies, respectively; of these, 7 and 9 events (cow-, calf-driven study) were removed due to poor camera angles interfering with observer ability to determine start or end times, or to confirm sucking. Following the removal of events <16 s in length (see 2.2.1 for explanation; events removed in cow-driven, calf-driven study: 356, 264), events occurring within 1 min between the same cow-calf pair – but that were previously separated by a short (i.e., <16 s) event on a different cow – were aggregated (cow-driven: 9 events; calf-driven: 12 events). This ensured that behavioral events followed the definitions as written in section 2.2, rather than being analyzed as separate events despite occurring on the same cow. One dam-calf pair was missing in the calf-driven study on the earliest observation period (i.e., age 3 weeks) due to treatment of the dam for mastitis in a sick pen.

#### 2.4.1 Suckling and allosuckling behavior

The 380 (cow-driven) and 419 (calf-driven) suckling bouts remaining after the initial data cleaning were further binomially classified as “suckling on dam” (0) or “allosuckling” (1) events. Prior to statistical analysis, the number of suckling bouts and total suckling time, regardless of whether performed on the dam or other cows, were summed per calf and day (defined here as a full, continuous 24-h period). Linear mixed effects models were then run, separately per study, using the lme4 package (33) with the following suckling behaviors as outcomes: daily suckling bouts (no. bouts/d), suckling bout duration (s/bout) and total suckling time (min/d). Fixed effects included in the models were average calf age (weeks; numeric) and bout type (0 = suckling on dam, 1 = allosuckling; suckling bout duration models only), while calf ID (cow-driven: n = 19; calf-driven: *n* = 24) was specified as a random intercept. Additionally, for models pertaining to the cow-driven study, calf sex was included as a fixed effect (no male calves in calf-driven study). All possible two-way interaction effects were tested but ultimately not included in the final models due to non-significance (*p* ≥ 0.05). Residuals were visually inspected to assess heteroscedasticity and normality for all models.

To explore possible factors related to allosuckling, we additionally used a generalized linear mixed model with a logit link function and binomial distribution [lme4 package (33)] for each respective study. In this case, the response variable was allosuckling (1/0). Model predictors included average calf age (weeks; numeric), calf sex (cow-driven study only), birth weight (kg) and presence of other suckling calves on the focal cow at the start of the bout (1/0), while calf ID (cow-driven: *n* = 19; calf-driven: *n* = 24) was included as a random intercept. Additionally, we wanted to explore factors associated with allosuckling when the dam was present in the cow-driven study, as dams could spend time in areas not accessible by calves. Therefore, the cow-driven dataset was first filtered to include only events where the dam was marked as present (*n* = 284 events). Then, a second generalized linear mixed model with logit link was run testing the same predictors (*n* = 19 calves), with allosuckling once again as the response, i.e., modeling the probability of allosuckling conditional on the dam being present. Log-odds estimates for all logistic regression models were transformed and reported as odds ratios.

Our literature review when planning the studies provided little evidence of breed influencing suckling behaviors in dairy calves, and as such breed was not included in our *a priori* hypotheses. However, since both our studies included two different breeds, additional exploratory *post hoc* analyses were performed including breed as a predictor. Results from these models are presented in Supplementary Tables 1, 2. The inclusion of breed resulted in only minor numerical changes in the estimates for the other predictors, with no effects on our main results.

Finally, we wanted to explore the relationship between the relative frequency of allosuckling per calf (% of all suckling bouts that were allosuckling) and ADG during the study period, as previous work on beef calves has suggested a slightly negative relationship between the two variables (34). Spearman’s rank correlation coefficients were calculated per study using correlation tests and are reported alongside *p*-values and correlation plots. Interpretation of correlation coefficients followed guidelines by Schober et al. (35).

#### 2.4.2 Suckling bout attributes

For each study, calf body position during suckling bouts, bout termination reason, and the occurrence of allogrooming were all descriptively reported separately for suckling bouts occurring on the dam and bouts of allosuckling. Data pertaining to allogrooming was not available for four of the recorded suckling bouts due to poor visibility of cow and/or calf head.

#### 2.4.3 Close-to-udder events

Following the initial data cleaning, the time (in min) from each close-to-udder event to the next suckling bout (for the same calf, on that same day) was calculated; this was not possible for all events (cow-driven: 31, calf-driven: 38) due to no more suckling bouts occurring during the observed time. The resulting data for the difference in time had a strong right skew; consequently, the median difference in time was calculated, and this value was used to categorize close-to-udder events as occurring shortly before the next suckling bout or not (see Supplementary Figures 1, 2).

The strong right skew of time to next suckling bout suggests that during many close-to-udder events, calves may have been actively seeking opportunities to suckle. To further explore this notion, we additionally evaluated if the frequency of close-to-udder events occurring close in time before the next suckling event was correlated with the frequencies of allosuckling bouts and suckling bouts on the dam. Correlation tests were performed to test all four possible associations and used to calculate Spearman’s rank correlation coefficients and corresponding *p*-values. Close-to-udder events were defined as occurring close in time if within the median time between close-to-udder events and subsequent suckling bouts.

## 3 Results

### 3.1 Suckling behavior

#### 3.1.1 Cow-driven study

Calves performed an average (SD) of 4 (1.5) suckling bouts per day, with no significant differences between sexes or as calves increased in age (Table 1). Similarly, the suckling bout duration did not change with calf age, but bouts of allosuckling were significantly shorter than suckling bouts between dam-calf pairs (LSMean ± SEM: 8 ± 0.6 vs. 12 ± 0.5 min/bout). Suckling bout duration and frequency did not differ significantly between male and female calves, but female calves tended to engage in more daily suckling than male calves (46 ± 2.9 vs. 36 ± 3.8 min/d). No effect of calf age was found for total daily suckling time, with calves spending an average of 42 (17.0) min/d engaged in suckling across the study period. Weekly average values for all suckling behaviors based on raw data can be viewed in Supplementary Table 3.

**Table 1:** Fixed-effect estimates (est.) and SE for all linear mixed effects models of suckling behavior in either a cow-driven (*n* = 19 dam-calf pairs) or calf-driven (*n* = 23 dams, *n* = 24 calves) CCC system.

Behavior	Cow-driven					Calf-driven				
	Est.	SE	*F*-value	df1, df2	*p*-value	Est.	SE	*F*-value	df1, df2	*p*-value
Total suckling bouts (bouts/d)										
Calf age	0.04	0.029	1.75	1, 75	0.19	−0.10	0.033	9.30	1, 70	0.003
Calf sex^1^	0.68	0.462	2.16	1, 17	0.16	–	–	–	–	–
ICC^2^	0.31					0.45				
Suckling bout duration (s/bout)										
Calf age	5.24	3.348	2.45	1, 362	0.12	24.53	3.541	47.92	1, 397	<0.001
Bout type^3^	−192.71	31.676	36.65	1, 376	<0.001	−177.59	41.121	18.44	1, 415	<0.001
Calf sex^1^	69.28	54.891	1.59	1, 18	0.22	–	–	–	–	–
ICC^2^	0.12					0.25				
Total suckling time (min/d)										
Calf age	0.38	0.357	1.11	1, 75	0.30	0.54	0.275	3.78	1, 70	0.06
Calf sex^1^	9.63	4.766	4.09	1, 17	0.06	–	–	–	–	–
ICC^2^	0.21					0.31				

Calf age (cow-driven study: 3–15 weeks, calf-driven study: 3–12 weeks) was included as a numeric variable, and bout type referred to suckling on dam vs. allosuckling. *p*-values are shown for main effects, and F-statistics and degrees of freedom were estimated using the Kenward-Roger method.

^1^Male calves were considered as the baseline; no male calves included in the calf-driven study.

^2^Intra-class correlation coefficient.

^3^Suckling bouts on dam were considered as the baseline.

#### 3.1.2 Calf-driven study

As calves aged, they changed their behavior to perform fewer suckling bouts per day (3 weeks: 5 ± 0.3 bouts/d, 12 weeks: 4 ± 0.3 bouts/d; Table 1). The duration of individual suckling bouts increased during this time, with bouts occurring on the dam being significantly longer than bouts of allosuckling at all ages (11 ± 0.5 vs. 8 ± 0.8 min/bout). Suckling bouts between dam-calf pairs increased in duration from 9 ± 0.6 min/bout at 3 weeks to 13 ± 0.6 min/bout at 12 weeks of age. There was a tendency for calves to spend more time suckling per day as they aged (3 weeks: 42 ± 2.0 min/d, 12 weeks: 47 ± 2.0 min/d), although this finding was not significant. For all suckling behaviors, weekly average values based on raw data can be viewed in Supplementary Table 3.

#### 3.1.3 Allosuckling behavior

**Figure 3:**
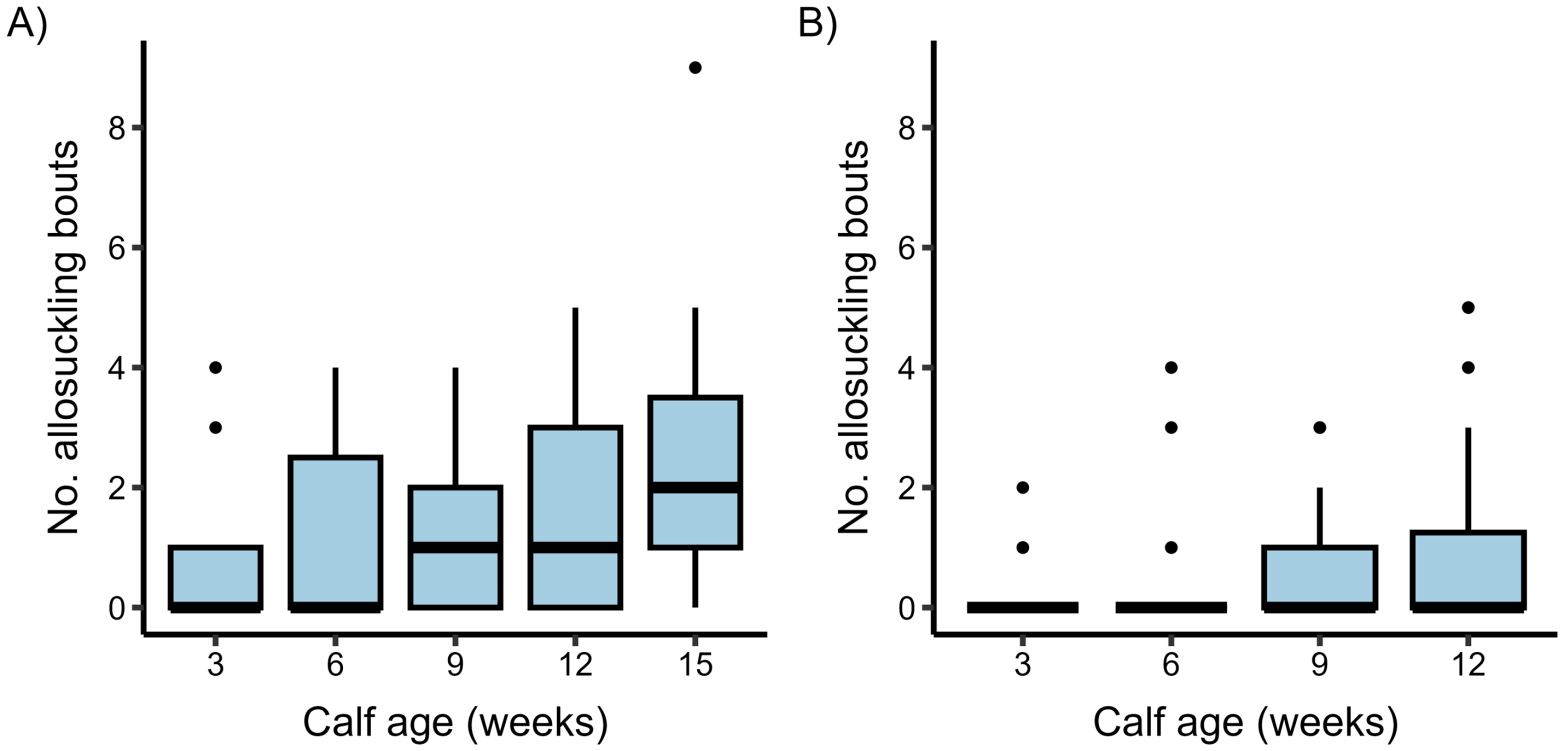
Boxplots presenting the number of allosuckling bouts per calf and observation day in a cow-driven **(A)** and calf-driven **(B)** CCC system across different ages. In the cow-driven study, dam-calf pairs (*n* = 19) could have full contact in a designated contact area within the pen, which cows could choose to leave at any time. For the calf-driven study, full contact between dams (*n* = 23) and calves (*n* = 24) was possible in all parts of the freestall pen. Box boundaries show interquartile range (IQR), whiskers represent 1.5 times the IQR, and the midline indicates the median value per calf age. Dots show values for all individual calves outside the whisker boundaries.

Out of a total 380 (cow-driven study) and 419 (calf-driven study) suckling bouts recorded, 36% and 14% were bouts of allosuckling in each study, respectively (see Figure 3 for a weekly breakdown). There were a number of calves that suckled exclusively on their own dams during the observation days in both studies, although this behavior was descriptively more prevalent in the calf-driven study (cow-driven: 2 calves, calf-driven: 12 calves). For the remaining calves (i.e., those that allosuckled at least once), the proportion of all suckling bouts that were performed on alien cows ranged from 8–61% (median: 40%) for the cow-driven study, and 4–61% (median: 15%) for the calf-driven study.

**Figure 4:**
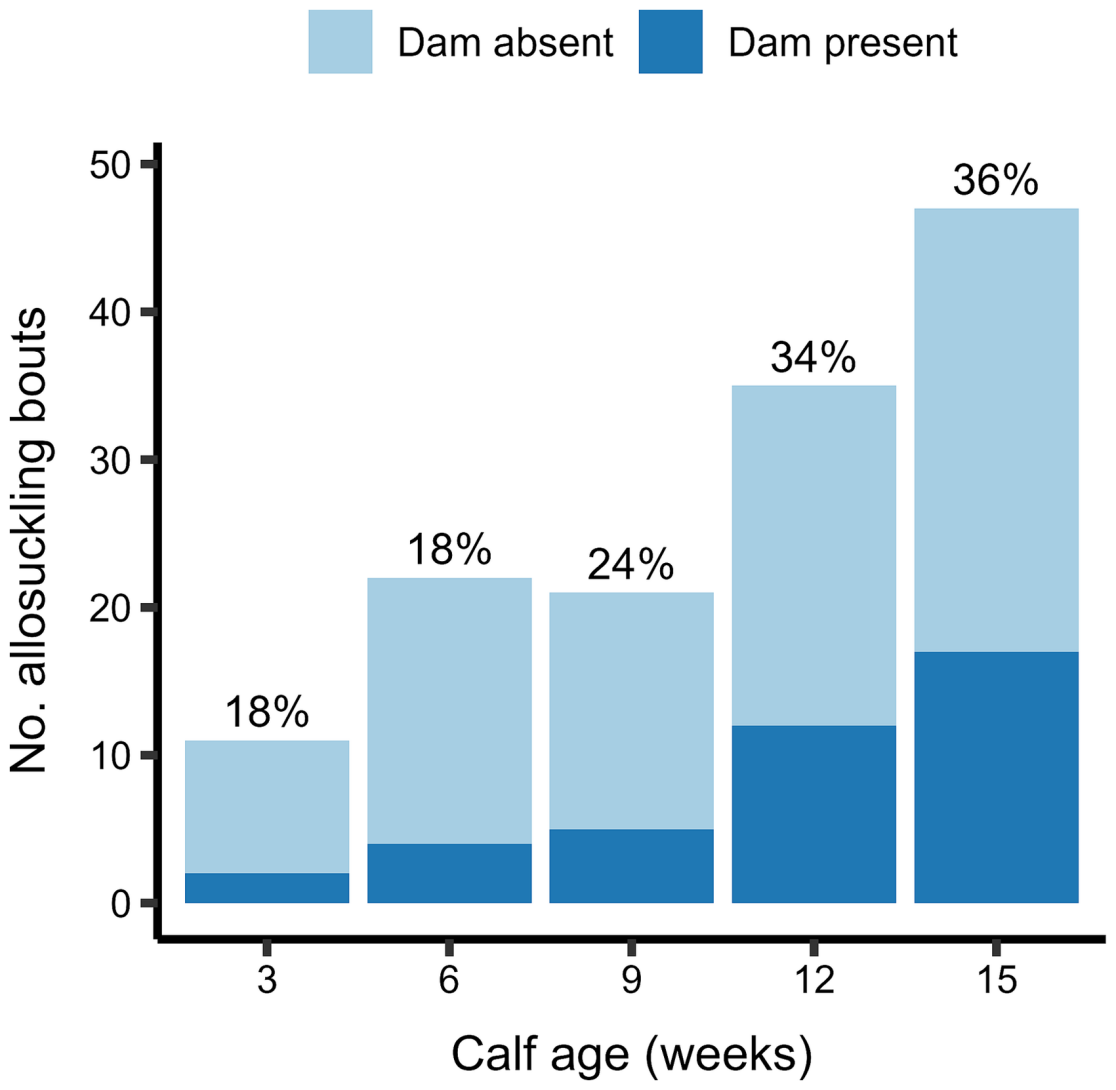
The total number of allosuckling bouts occurring at average calf ages of 3–15 weeks for calves (*n* = 19) housed in a cow-driven CCC system. At the start of each allosuckling bout, it was recorded whether the dam was present in the shared contact area – and thus physically available to the calf – or in a different area of the experimental pen. The proportion of bouts occurring with the dam present is shown as percentages above each bar.

The odds of allosuckling increased significantly with calf age in both the cow-driven and calf-driven studies (Table 2). In the cow-driven study, the odds of allosuckling increased by 4.4 from 3 to 15 weeks of age. The odds of allosuckling at 12 weeks in the calf-driven study were 7.3 times greater than the odds at 3 weeks of age. There was also a strong influence of other calves already suckling the focal cow on the odds of allosuckling, with 170 (cow-driven study) and 141 (calf-driven study) times higher odds of a calf successfully suckling on an alien cow if other calves were already suckling the same cow, compared to cases where that calf was the first to suckle. In 86% (cow-driven study) and 89% (calf-driven study) of occasions where a calf joined an alien cow already nursing at least one other calf, the cow’s own calf was among them. In general, allosuckling was more commonly observed as a group as opposed to solitary behavior, with calves suckling on an alien cow containing other suckling calves in 81% (cow-driven study) and 62% (calf-driven study) of all suckling events. Additionally, when the data set for the cow-driven study was filtered for suckling events occurring when the dam was present in the contact area, the odds of allosuckling increased as the calves grew older (Figure 4).

**Table 2:** Fixed-effect estimates, SE and *p*-values for all logistic mixed regression models of allosuckling behavior in either a cow-driven (*n* = 19 dam-calf pairs) or calf-driven (*n* = 23 dams, *n* = 24 calves) CCC system.

Behavior	Cow-driven			Calf-driven		
	Estimate	SE	*p*-value	Estimate	SE	*p*-value
Allosuckling (1/0)						
Calf age	0.12	0.046	0.01	0.22	0.074	0.003
Other calves	5.14	0.514	<0.001	4.95	0.795	<0.001
Calf birth weight	−0.04	0.039	0.27	0.03	0.065	0.68
Calf sex^1^	0.42	0.467	0.36	–	–	–
ICC^2^	0.01			0.40		
Allosuckling with dam present (1/0)						
Calf age	0.25	0.082	0.003	–	–	–
Other calves	5.59	0.950	<0.001	–	–	–
Calf birth weight	−0.12	0.077	0.13	–	–	–
Calf sex^1^	−0.27	0.908	0.77	–	–	–
ICC^2^	0.24			–		

Calf age (cow-driven study: 3–15 weeks, calf-driven study: 3–12 weeks) and birth weight were included as numeric predictors. Other calves refers to whether or not any non-focal calves were suckling the focal cow at the start of the focal suckling event and was scored binomially (1/0). Separate models were run for allosuckling in general and allosuckling only when the dam was present (i.e., physically available to the calf), which was not possible in the calf-driven study as the dam was always present.

^1^Male calves were considered as the baseline; no male calves included in the calf-driven study.

^2^Intra-class correlation coefficient.

**Figure 5:**
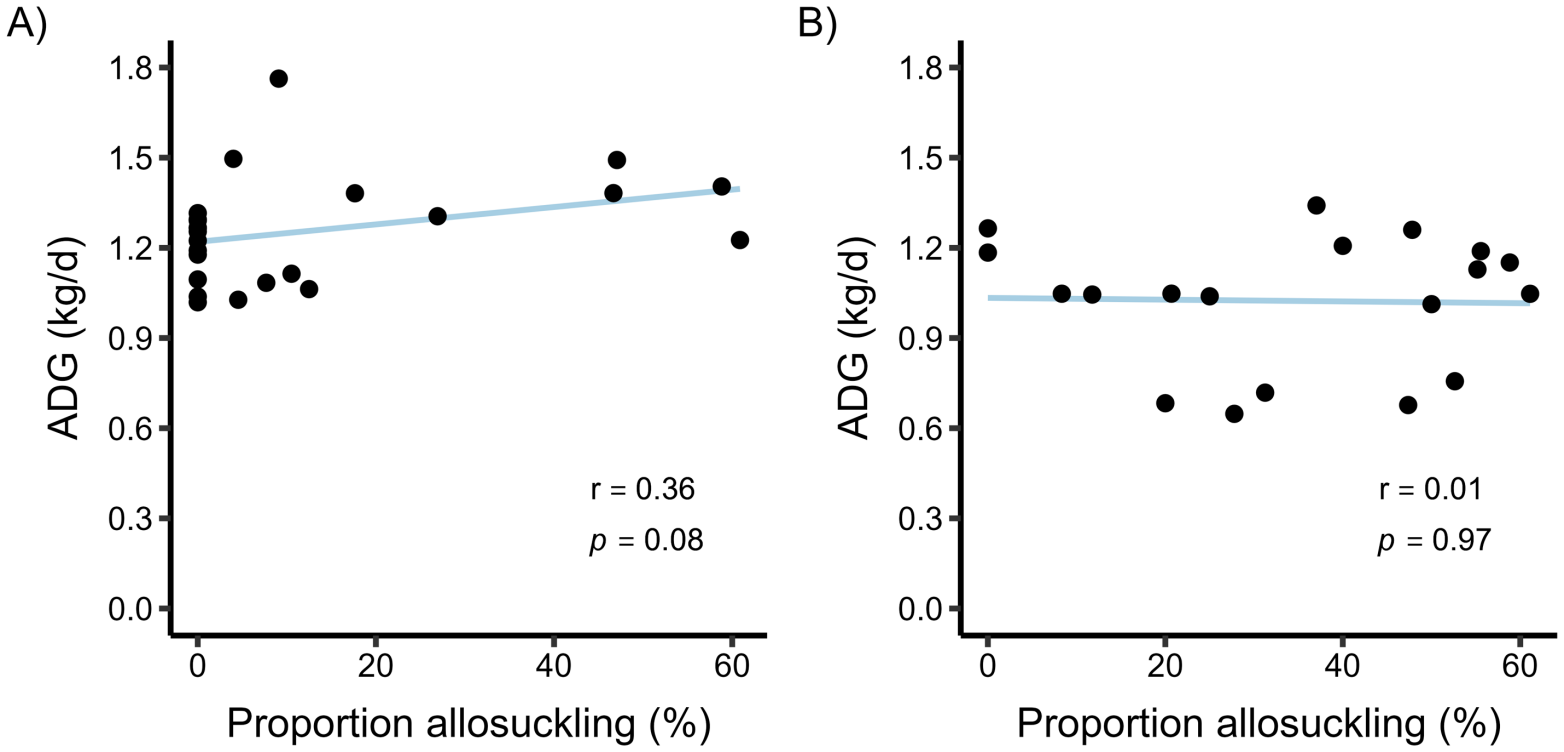
The association between average daily gain (ADG) and the proportion of allosuckling (% of all suckling bouts) per calf for a calf-driven **(A)** and cow-driven **(B)** CCC system. For the calf-driven study, full contact between dams (*n* = 23) and calves (*n* = 24) was possible in all parts of the freestall pen. In the cow-driven study, dam-calf pairs (*n* = 19) could have full contact in a designated contact area within the pen, which cows could choose to leave at any time. ADG was calculated using birth weight and body weight at an average (SD) calf age of 71 (11.3) and 104 (11.3) days for the calf-driven and cow-driven studies, respectively. Spearman’s Rank correlation coefficients (r) and *p*-values from correlation tests are displayed as text.

There was a tendency for a weak positive correlation between ADG throughout the study period and the relative frequency of allosuckling (% of all bouts that were allosuckling) for calves in the calf-driven study (Figure 5A). In the cow-driven study, no such correlation was found (Figure 5B).

### 3.2 Suckling bout attributes

When suckling on the dam, calves were primarily in an IP position (see Table 3). In contrast, allosuckling rarely occurred in this position, with calves instead positioning themselves perpendicular to or behind alien cows when suckling. Allogrooming occurred in 40% of bouts between dam-calf pairs in the cow-driven study, while it was observed only in 1% of allosuckling events. Similarly, in the calf-driven study allogrooming was observed in 49% of suckling bouts occurring on the dam, and during none of the allosuckling bouts.

**Table 3:** Percentage of suckling bouts performed in inverse parallel (IP), or from the side (S) or back (B) of the focal cow.

Study	Bout type	Total events	Calf body position (% of total events)		
			IP	S	B
Cow-driven	Suckling on dam	243	70	24	6
	Allosuckling	136	11	61	28
Calf-driven	Suckling on dam	361	86	9	5
	Allosuckling	58	15	47	38

Dam-calf pairs were freestall-housed with either cow-driven (*n* = 19 pairs) or calf-driven CCC (*n* = 23 dams, 24 calves) and observed for suckling behavior at average calf ages of 3, 6, 9, 12 (both studies) and 15 (cow-driven study only) weeks.

**Figure 6:**
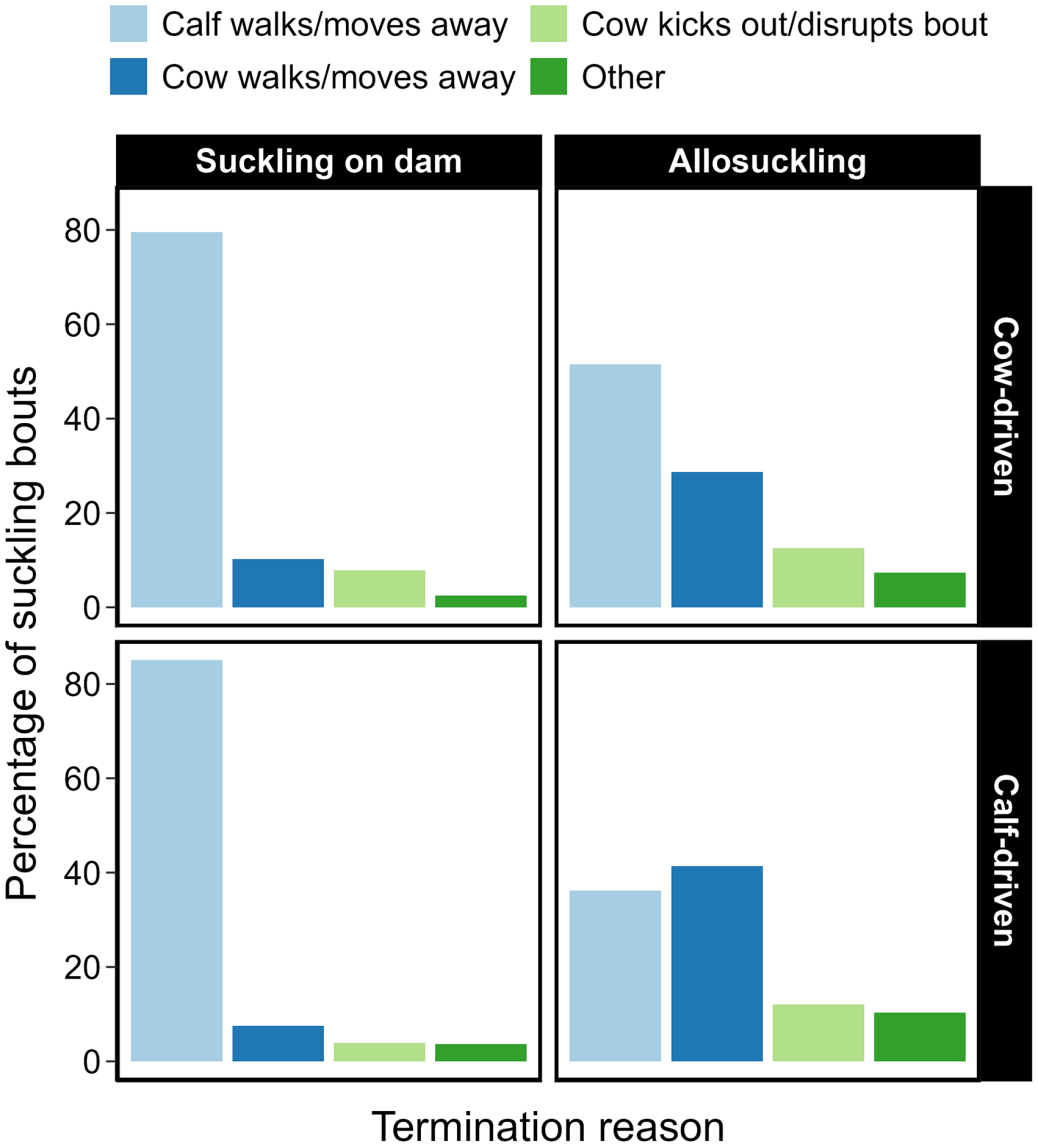
Reasons for termination of suckling bouts, displayed as percentages (of suckling on dam vs. allosuckling) for two types of CCC systems. Dam-calf pairs were either housed in a cow-driven CCC system (*n* = 19), where contact between pairs was only possible in a designated contact area, or in a calf-driven system, where CCC was possible throughout the entire pen for the included dams (*n* = 23) and calves (*n* = 24). Data is based on a total 799 suckling events collected across different days, corresponding to average calf ages 3, 6, 9, 12 (both systems) and 15 (cow-driven system only). The category ‘other’ includes bouts terminated by non-focal animals, barn staff, or equipment.

Suckling bouts between a dam and her calf were most often terminated by the calf (Figure 6). Conversely, approximately half of all allosuckling bouts (cow-driven study: 49%, calf-driven study: 53%) came to an end due to actions on part of the focal cow. The average duration of allosuckling bouts in the calf-driven study that were cow-terminated was numerically shorter than those terminated by calves, a pattern that was less pronounced in the cow-driven study (Table 4). Bouts ending due to kicking or other disruption (i.e., lunging, lying down or defecating) by the focal cow were, proportionally, quite similar between dam-calf pairs (8%) and unrelated cow-calf pairs (13%) in the cow-driven study. Meanwhile, in the calf-driven study, suckling bouts ending for this reason occurred more often in cases of allosuckling than for suckling on dam (12% vs. 4%).

**Table 4:** Mean (SD) duration of suckling bouts, per bout type (suckling on dam or allosuckling), as terminated by the cow, calf, or for another reason (e.g., bouts terminated by non-focal animals, barn staff or equipment).

Study	Behavior	Terminator of bout		
		Cow	Calf	Other
Cow-driven	Suckling bout on dam duration (min/bout)	12 (5.8)	11 (4.5)	12 (3.7)
	Allosuckling bout duration (min/bout)	9 (4.7)	10 (4.9)	7 (3.5)
Calf-driven	Suckling bout on dam duration (min/bout)	12 (6.0)	10 (4.4)	11 (5.1)
	Allosuckling bout duration (min/bout)	8 (4.0)	11 (5.6)	5 (2.8)

Dam-calf pairs were freestall-housed with either cow-driven (*n* = 19 pairs) or calf-driven CCC (*n* = 23 dams, 24 calves) and observed for suckling behavior at average calf ages of 3, 6, 9, 12 (both studies) and 15 (cow-driven study only) weeks. Mean values are based on raw values.

### 3.3 Close-to-udder events

A total of 233 (cow-driven study) and 265 (calf-driven study) close-to-udder events were recorded across the different calf ages. This behavior occurred between calves and their dams in 35% and 64% of events for the cow-driven and calf-driven study, respectively. The duration of close-to-udder events was most commonly very short (median [IQR]; cow-driven study: 48 [27–85] s; calf-driven study: 55 [26–101] s).

**Figure 7:**
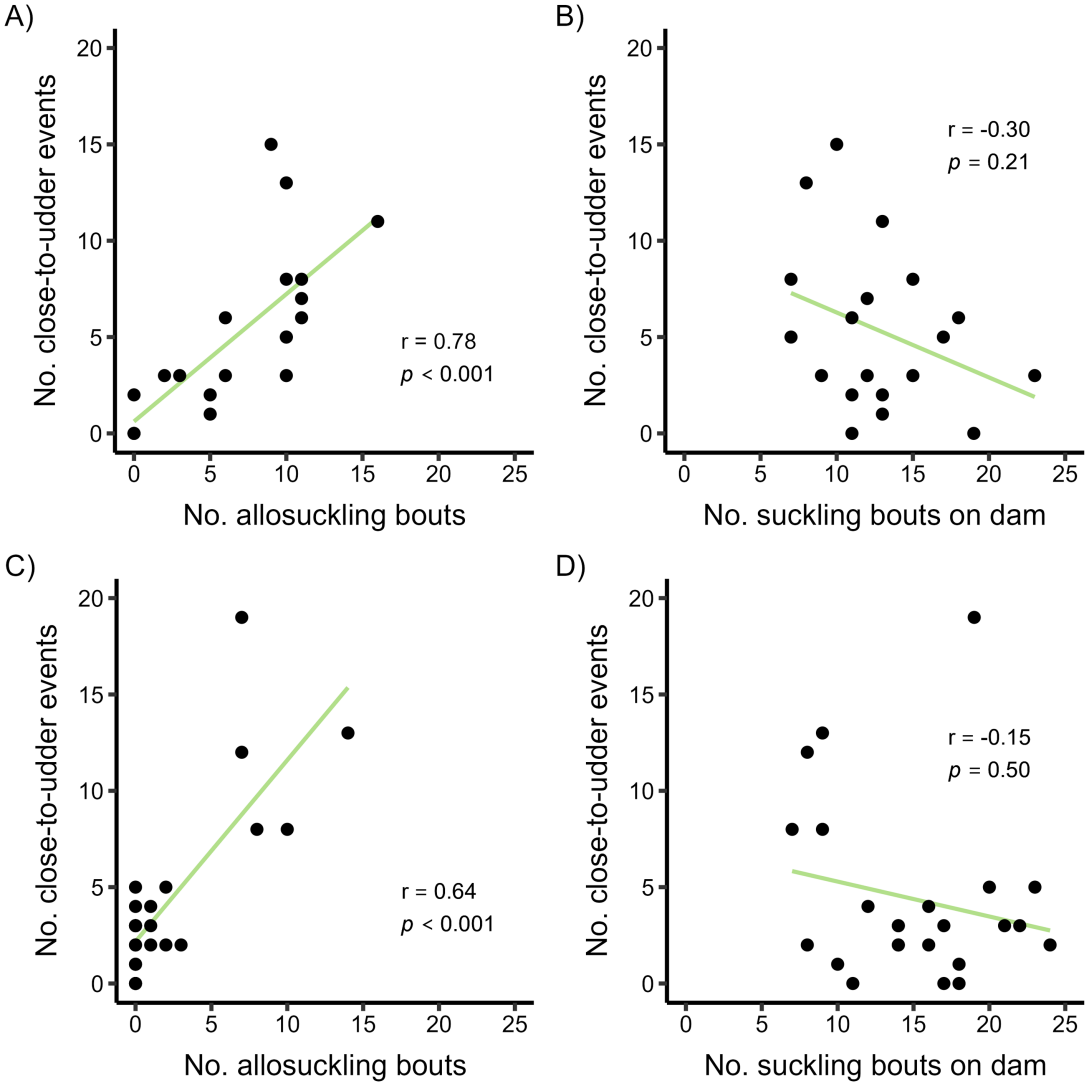
Correlation plots showing the frequency of close-to-udder events occurring shortly (defined as less than the median time difference) before the next suckling bout and the frequency of allosuckling bouts or alternatively number of suckling bouts on the dam per calf for a cow-driven **(A,B)** and calf-driven **(C,D)** CCC system. In the cow-driven study, dam-calf pairs (*n* = 19) could have full contact in a designated contact area within the pen, which cows could choose to leave at any time. For the calf-driven study, full contact between dams (*n* = 23) and calves (*n* = 24) was possible in all parts of the freestall pen. Spearman’s Rank correlation coefficients (r) and *p*-values from correlation tests are displayed as text.

Of the close-to-udder events in the cow-driven study, half occurred within 16 min of the next suckling bout (Supplementary Figure 1); these close-to-udder events were mainly terminated by cows (40%) or calves (56%), with a low number of events ending due to miscellaneous reasons. The number of close-to-udder events per calf occurring within 16 min of the next suckling bout was positively correlated with the number of allosuckling bouts performed by the calf during the study period (Figure 7A). Conversely, no correlation was evident between the frequency of close-to-udder events and suckling bouts performed on the dam (Figure 7B). As calves aged, close-to-udder events involving the dam decreased (59% at 3 weeks vs. 21% at 15 weeks), with calves instead directing this behavior toward alien cows to a higher degree.

The median time between close-to-udder events and subsequent suckling bouts in the calf-driven study was 71 min (Supplementary Figure 2). Of the events occurring within 71 min of suckling, the majority were terminated by calves (66%) as opposed to cows (30%). There was a moderate positive correlation between the number of allosuckling bouts per calf and number of close-to-udder events within 71 min of the next suckling bout, but it was less common for calves in the calf-driven study to allosuckle more than once (Figure 7C). In the calf-driven study, no correlation was found between the number of close-to-udder events and the frequency of suckling bouts involving dams (Figure 7D). Overall, close-to-udder events were primarily directed toward the dam in the calf-driven study, although the proportion decreased with increasing calf age (3 weeks: 84%, 12 weeks: 55%).

## 4 Discussion

In brief, calves in the cow-driven study allosuckled more frequently as they aged, but no other changes in suckling behavior were found. Calves in the calf-driven study performed fewer but longer suckling bouts as they aged, and allosuckling increased with age. While suckling behavior has previously been described for dairy calves housed together with their high-yielding dams indoors, we believe we are the first to do so beyond an average calf age of 9 weeks.

### 4.1 Suckling behavior of calves

Calves in the calf-driven study followed a pattern of behavioral change (i.e., fewer but longer suckling bouts) that aligns with expectations based on research of pastured beef (3, 6) and free-ranging Maremma (7) cattle. Similar age-related changes have been observed for dairy calves in various dam-rearing systems with whole-day contact. Calves housed with their dams in an indoor deep-bedded pack [calf ages: 2 & 4 weeks; (17)] or on pasture [ages: 3 & 6 weeks; (36)] suckled for longer durations as they grew older. Decreases in suckling bout frequency have also been reported from 3–8 weeks of age for calves in indoor CCC systems (14, 16). These changes in the frequency and duration of milk meals observed in other studies and our own may be, in part, due to the increasing stomach capacity of the calf as it ages. However, one question remains: Why were the same behavioral patterns not evident in the cow-driven study?

In the cow-driven study, neither the duration nor the frequency of suckling bouts was significantly influenced by calf age, although bout duration increased numerically between 3 and 12 weeks of age. One explanation is that perhaps the available time for contact – and thus, suckling – was more limited than in the calf-driven study. Johansson et al. (37) evaluated the time budgets of the dams in our cow-driven study, and reported that they spent on average at least 32% of their daily time budget outside the contact area (based on time spent on activities that could not have been performed in this area, e.g., milking and consuming forage in the feed alley). This would suggest that in terms of hours of dam-calf contact per day, the cow-driven study may have been closer to a half-day CCC system (i.e., 12 h/d), at least for some calves. Similar to our findings, Bertelsen and Jensen (16) reported that dairy calves reared with half-day CCC had no difference in the number of daily suckling bouts at 3 and 7 weeks of age, citing the restriction in contact time as the probable cause. The lack of changes in suckling behavior in our cow-driven study could thus be a sign of substantial restrictions in suckling time, potentially as a result of our pen set-up.

Alternatively, the lack of overall linear increase in suckling bout duration for this study may have been due to the numerically low value at 15 weeks of age. Since no obvious disruptions were noted in the barn during this observation period, it is unclear what may have caused suckling bouts on this day to be approximately 4 min shorter than at 12 weeks. One possibility is that the increase in this behavior for indoor-housed dairy calves is limited to the first 3 months of life, potentially due to increased suckling efficacy or greater solid feed intake beyond this point, but further investigation is needed to verify this notion.

In both the cow-driven and calf-driven study, allosuckling bouts were approximately 3–4 min shorter than suckling bouts performed on the dam. While comparisons of duration for bouts between calves and dams vs. alien cows have not been previously reported for dairy cattle, our findings are similar to that of beef calves kept on pasture (19) or indoors (38). Our results are logical if we consider that around half of all allosuckling bouts were terminated by the cow, and these bouts were often shorter than calf-terminated allosuckling bouts. As described further in section 4.3, allosuckling frequently occurred on cows that were already engaged in an ongoing nursing event, which in 86–89% of cases included the cow’s own calf. While cows that are nursing their own calves may be more tolerant of alien calves (14, 19), this tolerance likely dissipates once their calf has left.

Calves in the calf-driven study tended to spend more time suckling per day as they aged, likely due to the increasing bout duration. Meanwhile, daily suckling time in the cow-driven study remained stable with age. Only two previous studies have examined 24-h suckling time across different ages, and both reported no age effect (3, 24). However, these studies involved very young dairy calves [3–11 days old; (24)] or pasture-kept beef calves (3), limiting comparability with our findings.

Although age did not influence daily suckling time in the cow-driven study, female calves tended to spend more time suckling per day compared to male calves. Comparatively, other work has found no effect of calf sex on suckling behavior (14, 18). Although neither suckling bout frequency nor bout duration was statistically affected by calf sex, female calves had numerically more frequent and longer suckling bouts; hence, the combination of these two behaviors may have resulted in the greater daily suckling time for female calves.

Across all ages, the calves in both our studies performed approximately 4–5 suckling bouts/d, for 9–13 min/bout, which is within range of that reported by other studies that consider suckling within a 10-min period to be the same suckling bout (14, 24). Further direct comparisons of similarly-aged calves in literature are difficult due to differences both in study conditions and in suckling bout definitions; new bouts have been defined after pauses of anywhere between 3 s (15) and 2 min (19). Due to the definitions we used, it is likely that the bout durations and total suckling times reported in our own work are overestimated to an extent, as calves were occasionally noted to resume suckling bouts after relatively long pauses (i.e., nearly 10 min), and thus what we report as suckling bouts may closer represent suckling meals [see Špinka and Illmann (25)].

### 4.2 Allosuckling frequency in cow- and calf-driven CCC systems

As our two studies were performed in different pens, resulting in substantial differences in pen set-up and management, we were not able to statistically evaluate if allosuckling was affected by the type of CCC system. Descriptively, allosuckling was observed more frequently in the cow-driven study than in the calf-driven study (36 vs. 14% of all suckling bouts). In other recent work, calves in half-day CCC systems tended to be more likely to allosuckle compared to calves reared with whole-day CCC [ages: 3 & 7 weeks; (16)]. Johnsen et al. (15) similarly noted more frequent allosuckling when dams had restricted compared to free access to a contact area. If we continue the assumption that our cow-driven study more closely reflected half-day CCC, it is plausible that the calves in this study resorted to allosuckling if they were hungry when their dam was not present in the contact area.

Interestingly, Fröberg and Lidfors (14) reported a relative allosuckling frequency of only 16% for a cow-driven CCC system. This may be at least partially explained by less severe restrictions on contact time, as their contact area included all lying stalls within the experimental pen instead of only part of the lying area as in our cow-driven system. This highlights the importance of pen design for cow-driven CCC systems, as the direction of cow traffic and availability of shared resources (e.g., stalls) may influence the amount of time spent by cows in the contact area – and thus the amount of time available for calves to suckle and receive other maternal care.

In addition to its prevalence in other ungulate species [see review by Mota-Rojas et al. (39)], allosuckling has been reported for dairy calves across a variety of ages and systems (14–16, 18, 40, 41), as well as for indoor-housed beef calves (34, 38, 42), twin beef calves on pasture (19) and Zebu dairy calves with restricted suckling (10). Under extensive conditions, allosuckling in beef and Zebu cattle has been reported as non-existent (43), with attempts by calves being thwarted by dams from an early age (6). In contrast, dairy calves may find more success in allosuckling due to a selection for docility during milking (41, 44); indeed, few allosuckling bouts in our own studies ended due to kicking or lunging by the cow. It is also possible that dairy cattle are generally more accepting of alien calves; Loberg and Lidfors (45) reported that nearly all of 46 foster dairy cows permitted suckling by groups of four alien calves only using minimal human interference (e.g., tying up the cows). Yet without a direct comparison of dairy and beef breeds under matching circumstances, it is unclear if differences in allosuckling frequency are the result of differences in genetics, housing, management, or a combination of all three, since dairy breeds have not been evaluated for allosuckling under similarly extensive conditions as beef cows are typically kept.

Regardless, it is clear from both our own studies and those of others that allosuckling likely cannot be avoided in systems where dairy cows and calves are housed together. This raises the question: is allosuckling something we should strive to avoid? One potential concern with cows being suckled by multiple calves is that there is some evidence suggesting short-term damage to teats in dairy cows that were suckled by 3–4 calves for 15 min twice daily without additional milking (46). Given the study design, this finding may primarily be due to low milk yield of the cows combined with a high competition for teats, although it is unclear from available information if the calves were additionally supplemented with milk. Furthermore, the notion that calves may act as vectors for pathogen transmission between cows – and thus negatively impact udder health – remains unsubstantiated (47). Suckling by one or more calves can instead be beneficial for the dams in reducing the risk of mastitis, especially in early lactation, likely largely due to more complete udder emptying [see review by Beaver et al. (48)]. Yet not all dams are equally accepting of nursing alien calves, which is reflected in our work by the numerically higher proportion of allosuckling bouts (compared to suckling bouts between dam-calf pairs) that were terminated by the dam. In cow-driven CCC systems, cows have the possibility to physically remove themselves from situations of unwanted allosuckling by leaving the contact area, which dams in our cow-driven study were anecdotally noted to do on several occasions. In contrast, reprieve from calves was not possible in our calf-driven study; thus, from the perspective of cow welfare, calf-driven CCC may negatively impact dam agency.

Looking instead from a calf perspective, allosuckling may serve as a strategy to obtain adequate milk to maintain high growth (39), particularly in situations when dam access is limited. In both our studies, calves that frequently allosuckled had similar or slightly higher ADGs compared to calves that suckled more from their dams. Although the number of allosuckling bouts was numerically higher in the cow-driven study, the total daily time spent suckling and the frequency of suckling bouts were similar in both studies. This finding suggests that the calves in the cow-driven study were able to compensate for any restrictions in dam-calf contact through allosuckling. Ultimately, the question of how to weigh the benefits of allosuckling for calves against potential welfare consequences for dams (e.g., reduced agency) is beyond the scope of our research, but must be addressed as attention toward CCC systems continues to grow.

### 4.3 Calf-level factors associated with allosuckling

As the calves in our studies aged, we found that the odds of suckling on alien cows increased significantly, albeit to a numerically greater extent in the cow-driven study. This contrasts with recent work by Bertelsen and Jensen (16), who found that dairy calves were more likely to allosuckle at 3 versus 7 weeks of age. In beef calves, allosuckling has been reported to increase [calf age 1–100 d, (38); 2–5 mo, (19)] or remain constant [1–203 d, (34)] as calves age. It is unclear what exactly is driving this increase in the behavior in some settings. In our cow-driven study, allosuckling likely initially manifested primarily out of hunger, based on the high proportion of allosuckling bouts at ages 3 and 6 weeks that occurred when the dam was absent from the contact area. At the same ages, only a few calves in the calf-driven study were observed to allosuckle at all. Our findings in the calf-driven study align with other work indicating that young dairy calves prefer to suckle their own dam [i.e., <1 week old (25)].

One possibility for increased allosuckling is that as the calves grew older, those that had previously learned to allosuckle (e.g., out of hunger or opportunity) continued to do so at increasing frequencies, with each successful attempt reinforcing the behavior. Recent work indicates that dam-calf pairs form strong bonds even when suckling is prohibited (49), suggesting that from the calf’s perspective, a primary function of suckling is to provide it with nutrition, regardless of who acts as the provider (i.e., dam or alien cow). This might explain the increasing percentage of allosuckling observed in the cow-driven study even when the dam was present. While allogrooming may accompany suckling, this behavior was almost exclusively observed between dam-calf pairs in our studies, which aligns with the findings of others (7, 14) and suggests a separate motivation for this affiliative behavior than what motivates suckling.

Social factors may also to an extent explain the frequent observations of allosuckling. Increased intake of solid feeds has previously been attributed to social facilitation in group- (50) and pair-housed (51) calves, while pair-housed calves also demonstrate a higher frequency of milk-replacer meals than calves housed individually (52). In our studies, the odds of allosuckling were increased by over 140-fold when at least one other calf was already engaged in suckling. We deem it possible that the calves were socially influenced to start suckling when they saw and heard a suckling calf nearby, and often simply joined at the source of the milk (i.e., the cow already being suckled).

In the current studies, birth weight was not associated with allosuckling. Birth weight has previously been negatively associated with allosuckling frequency in beef and cross-bred calves, although this variable was interactive with the frequency of maternal suckling; more specifically, calves that weighed less at birth and suckled their dam less frequently were more likely to allosuckle (34). It is possible that in their study, calves with a low birth weight also had lower-producing dams, and thus sought milk elsewhere, as other work has suggested a positive relationship between birth weight of beef calves and milk supply of the dam (53). Furthermore, in our cow-driven study, no influence of calf sex on allosuckling was evident, aligning with work by Das et al. (10) on Zebu dairy calves in restricted suckling systems. In contrast, Víchová and Bartoš (34) noted higher frequencies of allosuckling in female versus male calves, although the authors themselves could not explain this finding.

Finally, there are likely calf-level factors beyond those explored in these studies that explain the degree of allosuckling observed for individual calves, as there were still a number of calves in both studies that were never observed to allosuckle. Though beyond the objectives of our studies, which focused on calf factors, specific dam-calf dyad factors and cow-level factors (e.g., parity, previous CCC experience) may also have influenced our findings. For example, an investigation that observes calves from the same cow, over different lactations, for similarity in allosuckling patterns in the offspring might clarify the dam’s contribution (if any) to this behavior.

### 4.4 Close-to-udder events

In the cow-driven study, close-to-udder events frequently involved alien cows and closely preceded suckling bouts; indeed, half of all close-to-udder events occurred within 16 min of an ensuing suckling bout, potentially representing feed-seeking in times of hunger. Considering that close-to-udder events in the calf-driven study often did not occur as close in time to the next suckling bout (median: 71 min), this lends support to our theory that calves in the cow-driven study at times were hungry when their dam was not available, and thus attempted to seek meals elsewhere. Moreover, the frequency of close-to-udder events occurring shortly (i.e., < 16 or 71 min) before the next suckling bout was found to be positively correlated with allosuckling frequency on calf-level in both studies, meaning that calves that frequently suckled on alien cows also often were scored as being very close to an udder without suckling shortly before their next successful suckling bout. Interestingly, only 30–40% of these close-to-udder events (i.e., occurring within 16 or 71 min of suckling) were recorded as terminated by cows. However, we urge caution in interpretation of the termination reasons, as they do not necessarily tell the whole story. For example, a cow may have kicked repeatedly at a calf suckling, but if the calf then made one last brief contact with the udder before walking away the event would be scored as calf-terminated.

Our intention with the recording of close-to-udder events was primarily to capture unsuccessful attempts at suckling, although the difficulty of detailed observations via video recordings from top-mounted cameras resulted in quite a broad definition of the behavior. As such, the nearly 500 close-to-udder events in our studies may also have included instances of sniffing or licking an udder, without the calf taking a teat into their mouth. As comparison, Fröberg and Lidfors (14), during direct observation, reported only 32 unsuccessful suckling attempts for 16 calves over seven different 24-h observation periods, likely due to their definition of suckling attempt including a teat being in the calf’s mouth. It would be interesting to see what fraction of our close-to-udder events – especially those close in time to suckling bouts – accurately represent attempts at suckling. For future studies, we would recommend using alternative observation methods (e.g., direct observations) that allow a refinement of our definition. Additionally, future research could consider combining observations of these two behaviors (i.e., close-to-udder events, suckling bouts) with data on other feeding behaviors; perhaps calves that learn to allosuckle (as an alternative food source to the dam) also begin to experiment with solid foods at an earlier age.

## 5 Conclusion

Dairy calves reared with cow-driven CCC did not alter their suckling patterns over time. Contrarily, calves with calf-driven CCC changed their behavior to perform fewer, but longer, suckling bouts as they aged. In both studies, the odds of allosuckling increased with calf age and were higher when performed in groups of at least two calves. However, allosuckling was more frequently observed among the calves with cow-driven CCC, even when their own dam was present. Our findings indicate that allosuckling can be expected in multiple types of dairy dam-rearing systems, although the extent to which it occurs may be related to calf-level factors and the duration of available daily contact time. This behavior may offer calves opportunities to satisfy their hunger when the dam is not available and thus maintain high growth during the contact period.

## Data Availability

The datasets presented in this study can be found in online repositories. The names of the repository/repositories and accession number(s) can be found below: Zenodo data repository, doi: 10.5281/zenodo.14778954.
