# Supplementary material for: Suckling and allosuckling behavior of dairy calves in indoor dam-rearing systems
Source: Front Vet Sci. 2025 Jul 17;12:1617158. doi: 10.3389/fvets.2025.1617158 (PMC12312680; doi:10.3389/fvets.2025.1617158)

Supplementary Material

Supplementary Table 1. Fixed-effect estimates (est.) and SE for all linear mixed effects models of suckling behavior in either a cow-driven (n = 19 dam-calf pairs) or calf-driven (n = 23 dams, n = 24 calves) CCC system. Calf age (cow-driven study: 3–15 weeks, calf-driven study: 3–12 weeks) was included as a numeric variable, and bout type referred to suckling on dam vs. allosuckling. *p*-values are shown for main effects, and F-statistics and degrees of freedom were estimated using the Kenward-Roger method.

	Cow-driven					Calf-driven				
Behavior	Est.	SE	*F*-value	df1,df2	*p*-value	Est.	SE	*F*-value	df1,df2	*p*-value
Total suckling bouts (bouts/d)										
Calf age	0.04	0.029	1.75	1,75	0.19	-0.10	0.033	9.26	1,70	0.003
Calf sex[1]	0.89	0.476	3.47	1,16	0.08	-	-	-	-	-
Calf breed[2]	-0.65	0.476	1.84	1,16	0.19	-0.87	0.437	3.93	1,22	0.06
ICC[3]	0.29					0.41				
Suckling bout duration (s/bout)										
Calf age	5.22	3.349	2.42	1,362	0.12	24.53	3.541	47.94	1,397	<0.001
Bout type[4]	-192.58	31.672	36.58	1,375	<0.001	-177.86	41.005	18.57	1,412	<0.001
Calf sex[1]	49.12	57.317	0.73	1,17	0.40	-	-	-	-	-
Calf breed[2]	61.06	56.557	1.16	1,16	0.30	105.07	60.920	2.97	1,21	0.10
ICC[3]	0.11					0.24				
Total suckling time (min/d)										
Calf age	0.38	0.357	1.11	1,75	0.30	0.54	0.275	3.80	1,70	0.06
Calf sex[1]	10.18	5.170	3.88	1,16	0.07	-	-	-	-	-
Calf breed[2]	-1.71	5.170	0.11	1,16	0.75	-1.39	3.223	0.19	1,22	0.67
ICC[3]	0.22					0.32				

[1]Male calves were considered as the baseline

[2]Swedish Red or Swedish Holstein, with the latter considered as the baseline

[3]Intra-class correlation coefficient

[4]Suckling bouts on dam were considered as the baseline

Supplementary Table 2. Fixed-effect estimates, SE and *p*-values for all logistic mixed regression models of allosuckling behavior in either a cow-driven (n = 19 dam-calf pairs) or calf-driven (n = 23 dams, n = 24 calves) CCC system. Calf age (cow-driven study: 3–15 weeks, calf-driven study: 3–12 weeks) and birth weight were included as numeric predictors. Other calves refers to whether or not any non-focal calves were suckling the focal cow at the start of the focal suckling event and was scored binomially (1/0). Separate models were run for allosuckling in general and allosuckling only when the dam was present (i.e., physically available to the calf), which was not possible in the calf-driven study as the dam was always present.

	Cow-driven			Calf-driven		
Behavior	Estimate	SE	*p*-value	Estimate	SE	*p*-value
Allosuckling (1/0)						
Calf age	0.12	0.046	0.01	0.22	0.074	0.003
Other calves	5.13	0.514	<0.001	4.99	0.804	<0.001
Calf birth weight	-0.04	0.038	0.30	0.02	0.063	0.71
Calf sex[1]	0.37	0.465	0.42	-	-	-
Calf breed[2]	0.19	0.418	0.64	0.45	0.829	0.59
ICC[3]	0.004			0.38		
Allosuckling with dam present (1/0)						
Calf age	0.25	0.083	0.002	-	-	-
Other calves	5.62	0.960	<0.001	-	-	-
Calf birth weight	-0.13	0.081	0.11	-	-	-
Calf sex[1]	-0.17	0.939	0.86	-	-	-
Calf breed[2]	-0.51	0.852	0.55			
ICC[3]	0.24			-		

[1]Male calves were considered as the baseline

[2]Swedish Red or Swedish Holstein, with the latter considered as the baseline

[3]Intra-class correlation coefficient

Supplementary Table 3. Mean (SD) from raw data of suckling behaviors of calves at different ages during an indoor suckling period with full CCC (cow-driven study: n = 19 calves; calf-driven study: n = 24 calves).

Behavior	Study	Calf age				
		3	6	9	12	15
Total suckling bouts (bouts/d)	Cow-driven	3.8 (1.30)	4.1 (1.05)	3.5 (1.02)	4.1 (1.66)	4.4 (2.04)
	Calf-driven	5.3 (1.66)	4.0 (1.16)	4.3 (1.37)	4.1 (1.33)	-
Suckling bout on dam duration (min/bout)	Cow-driven	11.1 (3.55)	11.8 (4.43)	12.0 (4.18)	13.8 (4.49)	11.1 (3.40)
	Calf-driven	9.0 (3.08)	11.1 (3.45)	11.5 (4.18)	13.1 (3.71)	-
Allosuckling bout duration (min/bout)	Cow-driven	8.8 (2.63)	8.3 (3.45)	10.4 (3.86)	11.5 (4.18)	8.4 (2.84)
	Calf-driven	5.2 (1.53)	11.6 (2.44)	9.5 (4.41)	7.3 (3.39)	-
Total suckling time (min/d)	Cow-driven	40 (13.3)	42 (13.5)	38 (13.2)	50 (25.4)	41 (15.5)
	Calf-driven	44 (12.4)	41 (9.1)	45 (11.2)	48 (9.8)	-

Supplementary Figure 1. Distribution of close-to-udder events based on how closely (in minutes) they preceded a suckling bout in a cow-driven cow-calf contact system. Data is based on close-to-udder events from 19 calves, and is separated based on events that involved dams (n = 67) vs alien (n = 135) cows. The horizontal grey line indicates the median time (16 min) across all data points.

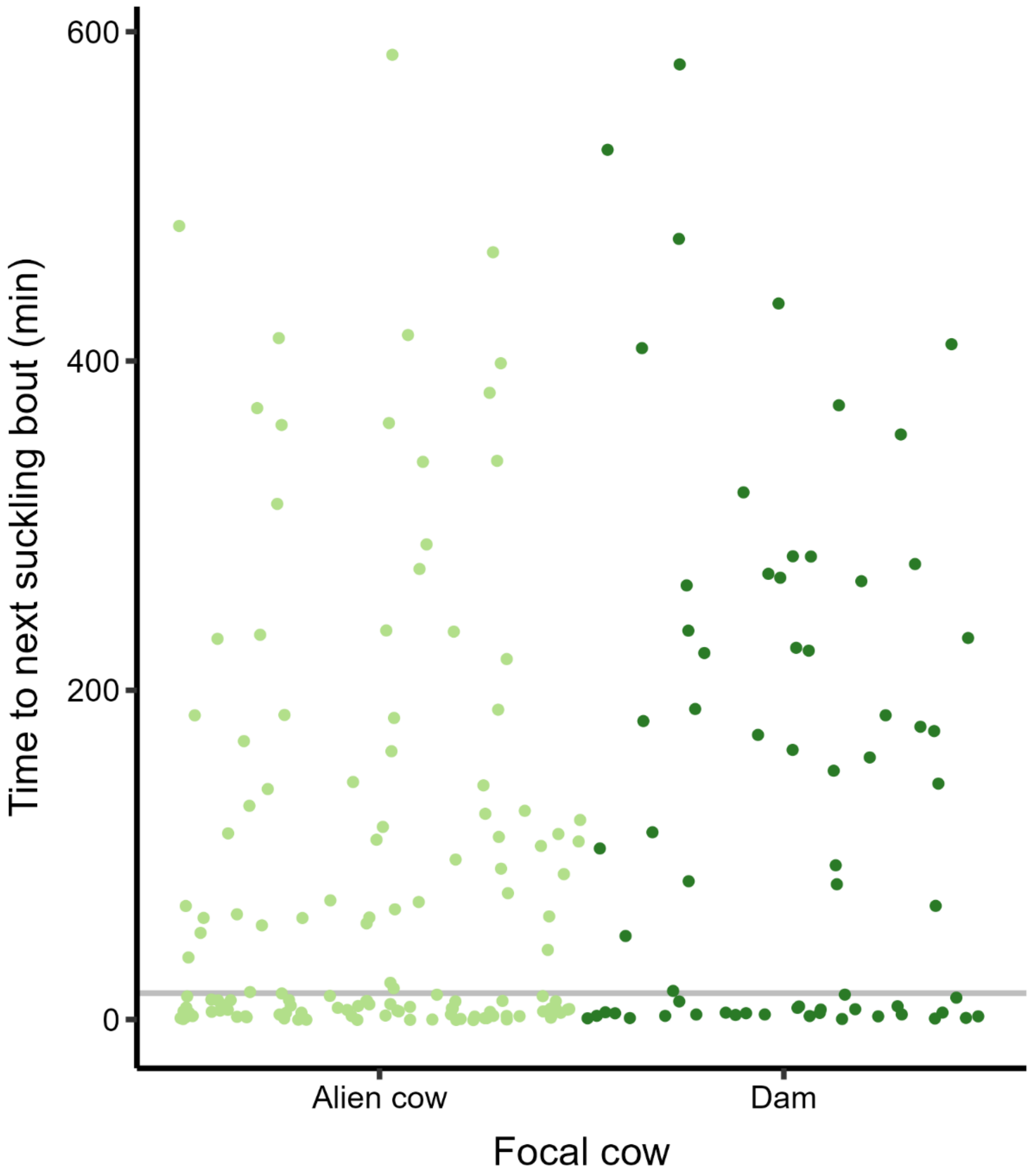

Supplementary Figure 2. Distribution of close-to-udder events based on how closely (in minutes) they preceded a suckling bout in a calf-driven cow-calf contact system. Data is based on close-to-udder events from 24 calves, and is separated based on events that involved dams (n = 128) vs alien (n = 82) cows. The horizontal grey line indicates the median time (71 min) across all data points.

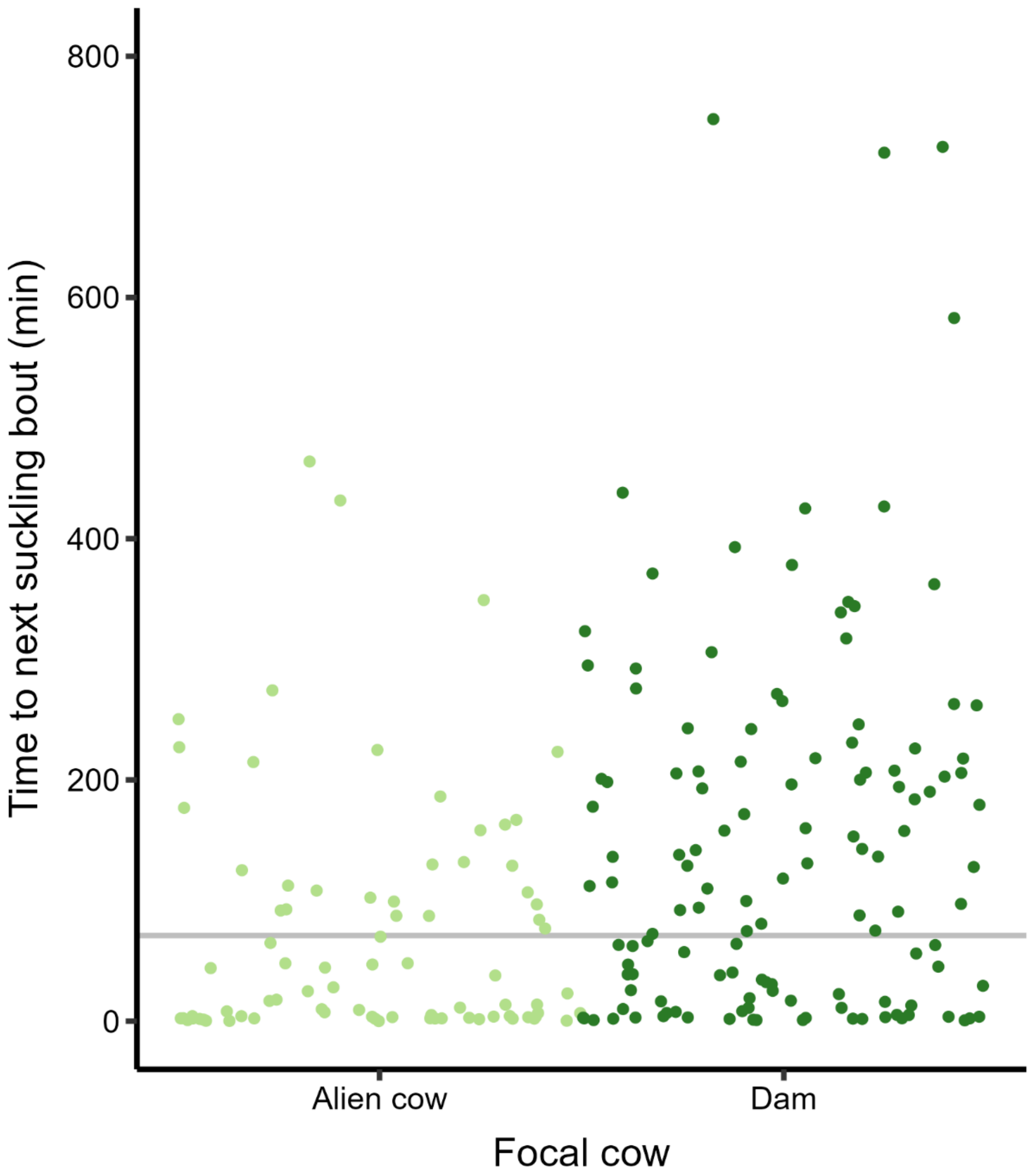

Supplement: Supplementary file 1 [file Supplementary_file_1.pdf]
